# Pregnancy planning, smoking behaviour during pregnancy, and neonatal outcome: UK millennium cohort study

**DOI:** 10.1186/1471-2393-13-238

**Published:** 2013-12-19

**Authors:** Anika Flower, Jill Shawe, Judith Stephenson, Pat Doyle

**Affiliations:** 1 King’s College London, School of Medicine, London, England; 2 Institute for Women’s Health, University College London, London, England; 3 London School of Hygiene and Tropical Medicine, London, England

**Keywords:** Pregnancy planning, Smoking, Low birthweight, Prematurity

## Abstract

**Background:**

Pre-pregnancy health and care are important for the health of the future generations. Smoking during pregnancy has been well-researched and there is clear evidence of harm. But there has been little research on the health impact of planning for pregnancy. This study aims to investigate the independent effects of pregnancy planning and smoking during pregnancy on neonatal outcome.

**Methods:**

This analysis made use of data from the UK Millennium Cohort Study. The study sample consisted of 18,178 singleton babies born in UK between 2000 and 2001. The neonatal outcomes of interest were low birthweight (<2.5 Kg) and pre-term birth (<37 completed weeks gestation). Logistic regression was used to estimate the association between pregnancy planning and/or smoking and neonatal outcome. Adjusted odds ratios were used to calculate population attributable risk fractions (PAFs).

**Results:**

43% of mothers did not plan their pregnancy and 34% were smoking just before and/or during pregnancy. Planners were half as likely to be smokers just before pregnancy, and more likely to give up or reduce the amount smoked if smokers. Unplanned pregnancies had 24% increased odds of low birth weight and prematurity compared to planned pregnancies (AOR_LBW_1.24, 95% CI 1.04-1.48; AOR_PREM_1.24, 95% CI 1.05-1.45), independent of smoking status. The odds of low birth weight for babies of mothers who were smoking just before pregnancy was 91% higher than that of mothers who were not (AOR_LBW_1.91, 95% CI 1.56-2.34). Women who quit or reduced the amount smoked during pregnancy lowered the risk of a low birth weight baby by one third (AOR_LBW_0.66, 95% CI 0.51-0.85) compared with women whose smoking level did not change. Smaller effects were found for prematurity. If all women planned their pregnancy and did not smoke before or during pregnancy, 30% of low birthweight and 14% of prematurity could, in theory, be avoided.

**Conclusions:**

Planning a pregnancy and avoiding smoking during pregnancy has clear, independent, health benefits for babies. Quitting or reducing the amount smoked during pregnancy can reduce the risk of low birthweight.

## Background

Pre-pregnancy health and care are important for the health of future generations. Growing evidence about the ‘fetal origins of adult disease’ [1] and from the field of epigenetics [2] indicate the large potential benefits of preconception and inter-conception care for both women and men. The life course approach to disease highlights the importance of the intrauterine environment in preventing future disease and the preconception period is seen as a critical period where intervention can lead to long term benefit [3]. The National Health Service (NHS) offers a range of guidance to women hoping to become pregnant [4]. Avoiding behaviour that can be detrimental to health such as smoking during pregnancy are highlighted in the NHS’s guidance. This is because smoking in pregnancy has a clear adverse impact on neonatal outcome, including preterm delivery, low birth weight, [5-7] still birth and up to 40% increased risk of infant mortality [8]. The National Institute for Health and Care Excellence (NICE) [9] offers public health guidance to health professionals for interventions to help women and partners to quit smoking including carbon monoxide testing and referral to smoking cessation services.

The NHS guidance also gives advice on how long it is expected for a woman to become pregnant and what options there are for fertility treatment if there are difficulties in becoming pregnant [4]. But there is no information on the health impact of pregnancy planning itself, reflecting the fact that there has been little research on decisions made by a woman before pregnancy and how that affects health related behaviours and the health of the child. Such information is important if interventions to improve neonatal outcome by preparing for pregnancy are to be initiated, and these interventions may need to be targeted towards particular groups who are less likely to plan for pregnancy.

This study uses Millennium cohort data to examine the independent effects of pregnancy planning and smoking during pregnancy on neonatal outcome. Specific objectives were to (i) examine the association between planning and smoking status, and (ii) examine the independent effects of pregnancy planning and smoking status on neonatal outcome, and (iii) estimate the proportion of adverse neonatal outcome that could be avoided in the population if all mothers planned their pregnancies and / or avoided smoking in pregnancy.

## Methods

### The millennium cohort study

The MCS is a nationally representative cohort study conducted by the Centre for Longitudinal Studies at the Institute of Education, London (http://www.cls.ioe.ac.uk/), that follows the lives of over 18,819 babies born throughout Great Britain and Northern Ireland between 2000 and 2001 [10] a&b. The MCS first collected information on the babies and their families from their two main carers (most commonly their mother and father) when the babies were around nine months old. The MCS attained an overall response rate of 68% throughout the whole of the UK [10] a&b. As part of the survey design the MCS oversampled in areas with high child poverty and in England also in areas with increased prevalence of ethnic minority populations [10] b.

### Pregnancy planning and smoking in pregnancy

The two main exposure variables were pregnancy planning and smoking status, which were asked about at the first interview when the child was 9 months of age. Pregnancy planning information came from the question to the mother “*Were you planning to get pregnant or was it a surprise*?” Women were grouped into those who planned, and those who did not plan, the pregnancy. Smoking status during pregnancy came from questions about current and past smoking, including the question “*How many cigarettes a day were you usually smoking just before you became pregnant*?” and “*Did you change the amount you smoked during your pregnancy*?” All women who reported a change had a reduction in the amount smoked. Women were classified as smokers just before pregnancy if they reported 1 or more cigarettes per day. For the analysis, four categories of smoking status during pregnancy were: (i) never-smoker; (ii) ex-smoker (given up before pregnancy); (iii) smoker just before pregnancy and had either quit smoking or had reduced the amount smoked during pregnancy; and (iv) smoker just before pregnancy and continued to smoke the same amount during pregnancy (no change).

### Neonatal outcome

The outcome variables were low birthweight (less than 2.5 Kg or 2.5 Kg and above) and pre-term birth (<37 completed weeks or 37 or more completed weeks gestation).

### Study population

This analysis investigated singleton births only. Records of babies from multiple births and corresponding carer information were dropped from the analysis (n = 522). A further 18 records were dropped from the analysis because the respondents were not immediate family members. 100 records had missing information on pregnancy planning, and 816 records had missing smoking information, leaving a total of 18,178 records with planning information and 17,462 records with smoking information. Further records (less than 145) with missing data on socio-economic characteristics and neonatal outcome were dropped from analyses, as appropriate: the numbers in each analysis are presented in the tables.

### Statistical analysis

Data for this analysis were taken from the first sweep only, accessed through the Economic and Social Data Service (http://www.esds.ac.uk/). All analyses used the statistical software package STATA, version 11. The survey design, oversampling and response rate were accounted for using the svyset command (and subsequent svy commands) in STATA using specific countrywide variables that had already been created.

Socio-demographic characteristics and the health status of women who planned, and did not plan, the index pregnancy were compared using descriptive tabulations and Chi square tests. Similar comparisons were made for women in the four smoking groups.

Univariable logistic regression was used to investigate the association between pregnancy planning, or smoking status, and neonatal outcome. Multivariable analysis was then conducted, including confounding variables in the model if they were associated with the outcome at a 5% level after adjustment for other factors in the model. Effect modification between planning and smoking status was investigated using Likelihood Ratio Tests (ignoring the SVY command). If no interaction was detected (p > 0.05) further adjustment was made for smoking status or planning, as appropriate.

Adjusted population attributable risk fractions (PAFs) were used to assess the independent impact of pregnancy planning and smoking on low birthweight and preterm delivery in this population. PAFs were computed using for formula:

$$\mathrm{PAF}\;\left(\%\right)\;=\;\frac{p\;\left(\mathrm{AOR}‒1\right)\;}{P\;\left(\mathrm{AOR}‒1\right)+1}\;x\;100\;$$

Where: p = proportion of population who did not plan their pregnancies/ or who smoked around the time of pregnancy, and AOR = Adjusted odds ratio.

PAFs to estimate the joint impact of pregnancy planning and smoking were calculated using the method described in Bruzzi et all, 1985 [11].

## Results

### Characteristics of the study population by planning and smoking status

Overall, 57% (10,405/18,178) of mothers of singleton births reported that they planned the pregnancy (Additional file 1: Table S1). Compared to planners, non-planners were younger and left school at an earlier age, were more likely to be classified as deprived, and less likely to be married and to have White ethnicity. The babies who were not planned were of higher birth order than babies who were planned and there was some indication that mothers who reported not planning their pregnancy were more likely to be underweight before pregnancy than women who did plan (Additional file 1: Table S1).

Fifty four percent (9,370/17,462) of mothers had never smoked and 12% (n = 2,071) were ex-smokers. Thirty four percent were smokers just before the pregnancy and, of these, 81% quit or decreased the amount they smoked during the course of the pregnancy (Additional file 2: Table S2). Compared to the non-smokers, smokers were younger and left school at an earlier age, were more likely to be classified as deprived, and less likely to be married. Again, there was some evidence that mothers who smoked around the time of the pregnancy were more likely to be underweight before pregnancy than women who did not smoke. Babies of mothers who continued to smoke in pregnancy had higher birth order than babies born to mothers in the other three smoking groups (Additional file 2: Table S2).

### Association between smoking and planning a pregnancy

There was a clear association between planning a pregnancy and not smoking just before pregnancy (p < 0.001, Table 1). Of the planners, 62% were never smokers, 12% ex-smokers and 26% were smoking just before pregnancy. The corresponding figures for mothers who did not plan their pregnancy were 46% never-smokers, 7% ex-smokers and 47% smoking just before pregnancy. Of those smoking just before pregnancy, 84% of the planners and 77% of the non-planners either quit smoking or reduced the amount they smoked in pregnancy (Table 1).

**Table 1:** Association between pregnancy planning status and smoking status

	**Smoking status just before pregnancy**				**Total n (%)**
	**Never smoked n (%)**	**Ex-smoker n (%)**	**Smoker: decreased or quit in pregnancy n (%) [*within smoker *%]**	**Smoker: no change in pregnancy n (%) [*within smoker *%]**	
**Mothers who planned pregnancy**	5,870 (62.3)	1,173 (12.4)	1,955 (21.1) [*82.1*]	425 (4.5) [*17.9*]	9,463 (100) [*100*]
**Mothers who did not plan pregnancy**	3,674 (45.9)	574 (7.2)	2,902 (36.2) [*77.1*]	860 (10.7) [*22.9*]	8,010 (100) [*100*]

Chi square for heterogeneity p < 0.001.

### Association between pregnancy planning, smoking in pregnancy, and neonatal outcome

Overall, 6% (1,102/18,178) of babies in the study population were born with low birthweight and 7% (n = 1,279) were born preterm.

#### Pregnancy planning

The proportion of low birthweight babies was lower for mothers who planned their pregnancy (5.2%) than for mothers who did not plan (7.2%). After adjusting for socio-demographic confounding factors (see table footnotes for details of adjustment factors) there was a statistically significant 27% increased odds of low birthweight for the children of mothers who did not plan compared to those who did (AOR 1.27, 95% CI 1.06-1.51)(Table 2). No effect modification between pregnancy planning and smoking was detected (p > 0.05). After further adjustment for smoking status, effect of planning reduced slightly to 24% increased odds (AOR 1.24, 95% CI 1.04-1.48), remaining statistically significant. Similarly, 6.3% of children whose mothers planned their pregnancy were born prematurely, compared to 8.2% of children whose mothers did not, with adjusted odds ratios showing 24% increased odds of prematurity associated with non-planning (AOR 1.24, 95% CI 1.05-1.45).

**Table 2:** Association between pregnancy planning status and neonatal outcome

	**Neonatal outcome**	**Neonatal outcome in**						
	**in mothers who**	**mothers who did not**	**Crude**^**1**^		Adjusted model 1 *		Adjusted model 2 **	
	**planned pregnancy**	**plan pregnancy**						
	**n/N (%)**	**n/N (%)**	**OR**	**(95% CI)**	**OR**	**(95% CI)**	**OR**	**(95% CI)**
**Low birth weight (<2.5 kg)**	541/10,394 (5.21)	561/7,762(7.22)	1.42	(1.22-1.65)	1.27	(1.06-1.51)	1.24	(1.04-1.48)
**Prematurity (< 37 weeks gestation)**	653/10,359 (6.30)	626/7,675 (8.16)	1.32	(1.15-1.52)	1.23	(1.05-1.45)	1.24	(1.05-1.45)

^1^The baseline group was women who had planned their pregnancy.

*All adjusted for: mother’s age, deprivation, relationship status, fertility treatment. Prematurity also adjusted for BMI.

**Adjusted for the same variables as model 1 plus smoking.

#### Smoking

The proportion of babies with low birthweight was higher for mothers who smoked just before pregnancy (8.3%) than for those who did not (5.0%). After adjustment for socio-demographic factors (see table footnotes for details) and pregnancy planning (no effect modification between smoking and pregnancy planning detected, p > 0.05), there was a statistically significant 91% increased odds of low birthweight associated with smoking before pregnancy (AOR 1.91, 95% CI 1.56-2.34) (Table 3). A smaller effect was seen for prematurity, the adjusted odds ratio showing 12% increased odds associated with smoking (AOR 1.12, 95% CI 0.95-1.35).

**Table 3:** Association between smoking before pregnancy (yes/no) and neonatal outcome

	**Neonatal outcome in mothers**	**Neonatal outcome in**						
	**who did not smoke before **	mothers who smoked*	**Crude**^**1**^		Adjusted 1 **		Adjusted 2***	
	**or during their pregnancy**	**before pregnancy**						
	**n/N (%)**	**n/N (%)**	**OR**	**(95% CI)**	**OR**	**(95% CI)**	**OR**	**(95% CI)**
**Low birth weight (<2.5 kg)**	569/11,429 (5.00)	501/6,017 (8.33)	1.73	(1.42-2.05)	1.98	(1.62-2.41)	1.91	(1.56-2.34)
**Prematurity (< 37 weeks gestation)**	748/11,352 (6.59)	485/5,966 (8.13)	1.25	(1.09-1.45)	1.14	(0.96-1.35)	1.12	(0.95-1.35)

^1^The baseline group was women who did not smoke during pregnancy.

*Including women who reduced or quit smoking at some time during pregnancy.

**All adjusted for mother’s age and mothers education. Birth weight is also adjusted for ethnicity, religion and BMI. Prematurity also adjusted for relationship status.

***Adjusted for the same variables as model 1 plus planning.

Further analysis of neonatal outcome for the children of mothers who reported smoking just before pregnancy showed that those mothers who quit or decreased the amount smoked during pregnancy had a lower proportion of low birthweight babies (7.6%) compared to mothers who continued to smoke the same amount in pregnancy (11.5%). After adjustment for confounding factors, including pregnancy planning, the odds of low birthweight for babies born to mothers who changed their smoking habits during pregnancy were reduced by 34% (AOR 0.66, 95% CI 0.51-0.85) (Table 4). A smaller and non-statistically significant effect of quitting or decreasing the amount smoked during pregnancy was seen for prematurity (AOR 0.85, 95% CI 0.67-1.09).

**Table 4:** Association between change in smoking behaviour during pregnancy (yes/no) and neonatal outcome, in mothers who smoked just before pregnancy

	**Mothers who smoked just before pregnancy**							
	**Neonatal outcome in mothers**	**Neonatal outcome in mothers**			**Crude**^**1**^		Adjusted 1*		Adjusted 2**	
	**who did not change their**	**who decreased or quit**							
	**smoking habit during pregnancy**	**smoking during pregnancy**							
	**n/N (%)**	**n/N (%)**	**OR**	**(95% CI)**	**OR**	**(95% CI)**	**OR**	**(95% CI)**	
**Low birth weight (<2.5 kg)**	136/1,183(11.50)	365/4,834(7.55)	0.62	(0.50-0.79)	0.65	(0.50-0.84)	0.66	(0.51-0.85)
**Prematurity (< 37 weeks gestation)**	109/1,167 (9.34)	376/4,799 (7.83)	0.82	(0.65-1.03)	0.85	(0.67-1.08)	0.85	(0.67-1.09)

^1^Baseline group was women who did not change their smoking habit during pregnancy.

*Birth weight adjusted for mother’s age, education, ethnicity, religion and BMI. Prematurity adjusted for mother’s age, relationship status and education.

**Adjusted for the same variables as model 1 plus planning.

### Population attributable risk fractions

**Figure 1:**
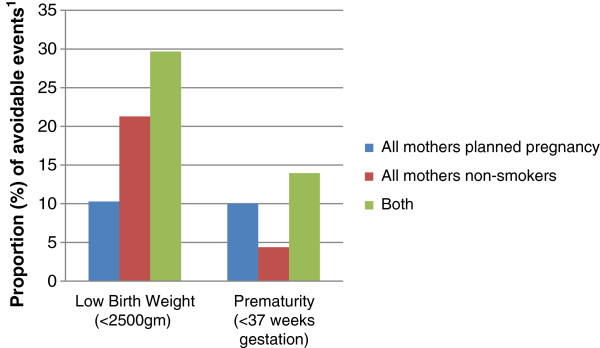
**Estimated proportion of adverse neonatal outcome which could theoretically be avoided in UK. **^1^Population Attributable Fraction (PAF) calculated using adjusted measures of effect (see methods).

Figure 1 presents the hypothetical proportion of adverse outcome that could be avoided if we assume that all women in this population planned their pregnancies, or no women smoked around the time of pregnancy, or both. Over 20% of low birthweight, and 4% of prematurity could have been avoided if no women smoked during and/or just before pregnancy. For pregnancy planning, the potential saving was a further 10% of both low birthweight and prematurity. The combined savings if all women planned and no women smoked, was almost 30% of low birthweight and 14% of prematurity.

## Discussion

### Main findings

Not surprisingly, those women who planned their pregnancy were less likely to smoke than those for whom the pregnancy was a surprise. However, there was strong evidence for an association between planning a pregnancy and neonatal outcome which was independent of smoking status. Surprise pregnancies had almost one quarter increased risk of low birth weight and prematurity compared to pregnancies which were planned after allowing for differences in smoking. Smoking just before pregnancy had a stronger association with low birthweight, smokers having over 90% increased risk of low birthweight compared to non-smokers. For those who were smoking just before pregnancy, giving up or reducing the amount smoked had a clear beneficial effect on low birthweight, risks being reduced by just over one third. These effects were independent of planning status and other health behaviours measured in this cohort. We estimated that, in theory, 30% of low birthweight, and 14% of premature births, could have been avoided in this population if all women planned their pregnancies and were non-smokers around the time of pregnancy.

### Strengths and limitations

This analysis used data from a large, nationally representative, UK cohort study. The size provided the study with good statistical power and the data contained information on potential confounding factors with a low proportion of missing information. However, as the information was collected nine months post birth there is likely to be some information bias as respondents were asked to recall events up to two years before they were interviewed. Self-reported smoking habits, in particular, is vulnerable to underreporting [12]. Although we are reassured to some extent that the rates of pre-pregnancy smoking were similar to national rates for 2000 reported elsewhere [13], we need to assess the impact of underreporting of smoking during pregnancy on our findings. If under reporting of smoking during pregnancy was similar in those with and without a low birthweight or preterm baby the effect would be to bias the measure of effect towards a null effect. It is thus unlikely to be an explanation for the smoking effects seen in these data. However, since the outcome of pregnancy was known at the time the questions on smoking were asked, we have to consider the possibility that underreporting of smoking status during pregnancy could be different for those with, and without, a low birthweight/preterm baby. For differential under-reporting of smoking habits to explain the findings reported here, under-reporting would have to be less likely (reporting being more accurate) for mothers with low birthweight/preterm babies compared to that for mothers with babies not low birthweight/preterm. We have no way of checking this with available data, and differential under-reporting of smoking during pregnancy may explain some of the effects reported here.

The MCS also only obtained information on babies that had survived to roughly nine months. A subsequent investigation estimated that 180 babies did not survive to nine months of age and so were not able to be surveyed [14]. Four percent of records also had missing smoking data. Since death and smoking are related to lowbirthweight and prematurity, missing data of this type may have resulted in an underestimation of the effect of smoking on lowbirthweight and smoking in this analysis.

Part of this research compared smokers who changed their behaviour and those who did not. There may have been some women in the no change group who continued to smoke a relatively little amount, and those in the group that changed their smoking behaviour who still smoked a comparatively large amount, for example if they cut down from 40 cigarettes per day to 10. We did not examine amounts smoked in this analysis. However, such misclassification of exposure would tend to bias the measure of effect towards the null, making our findings conservative rather than inflated.

### Interpretation

The characteristics of women in the MCS who planned, or did not plan, their pregnancies, and who smoked, or not, just before pregnancy has been reported before [15,16]. As expected, we found similar socio-economic distributions for these sub-groups within the MCS population. However, what is unique in the current - analysis is examination of the relationship between planning and smoking, and their independent effects on neonatal outcome.

While it may seem intuitive that unplanned pregnancies would be associated with poorer neonatal outcomes, the literature on this question is not extensive and is somewhat conflicting. One of the main reasons for this is the issue of adjustment for confounding factors including a range of health-seeking behaviours that may be associated with pregnancy planning but independently related to birth outcomes, such as good antenatal care.

Studies of women who plan compared to those who do not have investigated the effect on health related behaviours such as smoking, alcohol consumption, folic acid supplementation and antenatal care attendance. A study of Turkish - pregnant women interviewed in an antenatal setting found that 71% planned their pregnancy and they were less likely to smoke, have lower alcohol consumption, go to more antenatal sessions and take more supplementation and nutrition compared to those who did not plan [17]. The effect of planning a pregnancy appeared beneficial in this study, but a Swedish study found only 20% of women planning their pregnancy took folic acid during the period in which they were planning [18]. A Canadian study found a similar level of 28% of women who planned took folic acid supplementation [19]. In both the latter two studies, folic acid supplementation was more common in those who planned, compared to those who did not plan, their pregnancy, but the percentage falls far short of optimum coverage of folic acid supplementation before and during pregnancy.

Green-Raleigh et al found that women who were planning to get pregnant were more likely to decrease their consumption, or abstain, from alcohol [20]. The study also found that women planning to get pregnant were less likely to be smokers, [20] as we found in the current study. Another study which looked at the effect of planning in adolescents, found that those who planned their pregnancies had a higher rate of smoking, STDs, leaving school and subsequent pregnancies compared to those who did not plan [21]. It is thus unclear whether the positive effect of planning on health behaviour transcends across different age groups or if such decisions may affect subsequent changes in health related behaviour.

There has been surprisingly little research into the effect of pregnancy planning on morbidity in pregnancy but unplanned pregnancy, severe pregnancy related nausea and vomiting, high perceived stress and low social support were found to be associated with lower levels of psycho-social adjustment during pregnancy in a Taiwanese study [22]. A study in Turkey also found women who did not plan their pregnancies had higher rates of depressive symptoms during their pregnancy [23].

Research from as early as the 1970s showed that smoking in pregnancy increases the risk of having a low birth weight baby [24]. Our findings add to this evidence-base, demonstrating a clear impact of smoking on low birthweight after adjustment for confounding factors. Smoking during pregnancy has also been shown to increase the risk of having a baby that is small for gestational age and suffering from fetal growth restriction [25-27]. The impact on the fetus’ growth may be partly explained by the increased incidence of abnormal placental structure and function related to smoking [28]. A review of intervention studies has also shown that smoking cessation during pregnancy can reduce the risk of low birth weight and pre-term birth [29].

## Conclusions

Evidence about the adverse effect of smoking before and during pregnancy is strong, and effective smoking cessation interventions have been identified. Our study provides additional evidence of the benefits of stopping or reducing smoking during pregnancy. The message that it is never too late to give up smoking needs to be emphasised, especially at this critical time and for disadvantaged women who are more likely to smoke during pregnancy.

With over 40% of pregnancies in this study being a surprise, a more targeted policy to increase pregnancy planning and awareness of pre-pregnancy health is recommended. Compared to pregnancy planners, non-planners tended to be young, single and less well educated women. However, more rigorous measurement of pregnancy planning is needed to establish the full impact of pregnancy planning on neonatal outcomes.

## Abbreviations

MCS: Millenium Cohort Study; AOR: Adjusted odds ratio.

## Competing interests

The authors declare that they have no competing interests.

## Authors’ contributions

AF developed the study objectives and design, obtained the data, undertook analyses and wrote the first draft of the paper. JS suggested the study idea and source of data, checked the analyses and contributed to the writing of the paper. JS contributed to the study design, checked the analyses and contributed to the writing of the paper. PD developed the study objectives and design, checked data, undertook analyses, and made a major contribution to the writing the paper. All authors read and approved the final manuscript.

## Pre-publication history

The pre-publication history for this paper can be accessed here:

http://www.biomedcentral.com/1471-2393/13/238/prepub

## Supplementary Material

Additional file 1: Table S1 Socio-demographic and health status characteristics of women in the study population overall and separately for those who planned and did not plan their pregnancy. Click here for file

Additional file 2: Table S2 Socio-demographic and health status characteristics of women in the study population overall and separated by their smoking behaviour. Click here for file
