# Supplementary material for: Pregnancy planning, smoking behaviour during pregnancy, and neonatal outcome: UK millennium cohort study
Source: BMC Pregnancy Childbirth. 2013 Dec 19;13:238. doi: 10.1186/1471-2393-13-238 (PMC3878353; doi:10.1186/1471-2393-13-238)
Supplement: Additional file 2: Table S2 — Socio-demographic and health status characteristics of women in the study population overall and separated by their smoking behaviour. [file 1471-2393-13-238-S2.doc]

| **Additional file 2: Socio-demographic and health status characteristics of women in the study population overall and separated by their smoking behaviour** | | | | | | | | | | | |
| --- | --- | --- | --- | --- | --- | --- | --- | --- | --- | --- | --- |
|  | Smoking status just before pregnancy | | | | | | | | P-value |  |  |
|  | Never Smoked | | Ex-smoker | | Smoker: Decreased or Quit in Pregnancy | | Smoker :No Change in Pregnancy | |  | Total |  |
|  | n | % | n | % | n | % | n | % |  | n | % |
|  | 9,370 | 53.66% | 2,071 | 11.86% | 4,839 | 27.71% | 1,182 | 6.77% |  | 17,462 | 100.00% |
| **Mother’s Age:** | |  |  |  |  |  |  |  | |  |  |
| 13-19 | 382 | 4.08% | 40 | 1.93% | 728 | 15.04% | 159 | 13.45% | <0.0001 | 1,309 | 7.50% |
| 20-29 | 3,855 | 41.16% | 754 | 36.43% | 2,528 | 52.24% | 616 | 52.12% |  | 7,753 | 44.41% |
| 30-39 | 4,881 | 52.12% | 1,206 | 58.26% | 1,531 | 31.64% | 384 | 32.49% |  | 8,001 | 45.84% |
| 40 Plus | 248 | 2.65% | 70 | 3.37% | 52 | 1.07% | 23 | 1.95% |  | 395 | 2.25% |
| **Relationship Status:** | |  |  |  |  |  |  |  | |  |  |
| Married / Co-habiting | 8,493 | 90.76% | 1,926 | 93.53% | 3,548 | 73.50% | 785 | 67.27% | <0.0001 | 14,752 | 84.65% |
| Separated/ Divorced | 106 | 1.13% | * | - | 54 | 1.12% | * | - |  | 185 | 1.06% |
| Closely Involved | 432 | 4.62% | 70 | 3.41% | 603 | 12.49% | 173 | 14.82% |  | 1,278 | 7.33% |
| Just Friends/No relationship | 327 | 3.49% | 54 | 2.63% | 622 | 12.89% | 209 | 17.91% |  | 1,212 | 6.95% |
| **Birth order of index child:** | | |  |  |  |  |  |  | |  |  |
| First born | 3,943 | 42.08% | 718 | 34.67% | 2,380 | 49.17% | 332 | 28.09% | <0.0001 | 7,373 | 42.22% |
| Second born | 3,405 | 36.34% | 920 | 44.42% | 1,516 | 31.32% | 426 | 36.04% |  | 6,267 | 35.89% |
| Third born or later | 2,023 | 21.58% | 433 | 20.91% | 944 | 19.50% | 424 | 35.88% |  | 3,824 | 21.89 |
| **Deprivation (based on mothers postcode):** | | | | | | | | | | | |
| Above 60% (less deprived) | 6,710 | 77.96% | 1,621 | 83.90% | 2,677 | 58.76% | 461 | 42.80% | <0.0001 | 11,469 | 70.92% |
| Below 60%(More deprived) | 1,897 | 22.04% | 311 | 16.10% | 1,879 | 41.24% | 616 | 57.20% |  | 4,703 | 29.08% |
| **Mother’s Education (Age left School):** | | | | | | | | | | |  |
| Still in School | 63 | 0.68% | * | - | * | - | * | - | <0.0001 | 82 | 0.47% |
| Left at 16 or under | 3,404 | 34.48% | 858 | 41.49% | 2,995 | 62.15% | 942 | 80.03% |  | 8,196 | 47.07% |
| Left at 17 or 18 | 2,932 | 31.42% | 651 | 31.48% | 1,242 | 25.77% | 174 | 14.18% |  | 4,999 | 28.71% |
| Left at 19 or older | 2,932 | 31.42% | 559 | 27.03% | 582 | 12.08% | 61 | 5.18% |  | 4,134 | 23.74% |
| **Ethnicity:** | | | | | | | | | | | |
| White | 7,654 | 81.84% | 1,989 | 98.81% | 4,595 | 95.19% | 1,139 | 100% | <0.0001 | 15,377 | 88.28% |
| Mixed | 70 | 0.75% | * | - | 70 | 1.45 | * | - |  | 165 | 0.95% |
| Indian or Pakistani | 1,036 | 11.08% | * | - | 40 | 0.83% | * | - |  | 1108 | 6.36% |
| Black or Black British | 362 | 3.97% | * | - | 78 | 1.62% | * | - |  | 481 | 2.76% |
| Other | 240 | 2.46% | 24 | 1.19% | 44 | 0.91% | * | - |  | 287 | 1.65% |
| **Religion:** | | | | | | | | | | | |
| No religion | 3,372 | 36.10% | 940 | 45.95% | 2,902 | 60.58% | 752 | 65.05% | <0.0001 | 7,966 | 45.79% |
| Roman Catholic | 960 | 10.28% | 270 | 13.20% | 552 | 11.52% | 137 | 11.85% |  | 1,919 | 11.03% |
| Church of England | 2,224 | 23.81% | 524 | 25.62% | 823 | 17.18% | 177 | 15.31% |  | 3,748 | 21.54% |
| Christian Other | 1,455 | 15.58% | 285 | 13.94% | 473 | 9.87% | 90 | 7.79% |  | 2,303 | 13.24% |
| Muslim/Islam | 959 | 10.27% | 26 | 1.27% | 40 | 0.84% | * | - |  | 1,041 | 5.98% |
| Other | 371 | 3.97% | * | - | * | - | * | * |  | 421 | 2.42% |
| **Diabetes in pregnancy:** | | | | | | | | | | | |
| No | 9,191 | 98.08% | 2,025 | 97.83% | 4,749 | 98.19% | 1,170 | 100% | 0.2088 | 17,137 | 98.21 |
| Yes | 180 | 1.92% | 45 | 2.17% | 87 | 1.81% | * | - |  | 323 | 1.79% |
| **Pre-pregnancy Body Mass Index (self reported):** | | | | | | | | | | | |
| Underweight (<18 kg/m^3^) | 366 | 4.17% | 58 | 2.92% | 368 | 8.00% | 118 | 10.68% | <0.0001 | 910 | 5.53% |
| Normal | 5,831 | 66.44% | 1,306 | 65.79% | 3,091 | 67.18% | 661 | 59.82% |  | 10,889 | 66.12% |
| Overweight | 1,777 | 20.25% | 427 | 21.51% | 816 | 17.74% | 229 | 20.72% |  | 3,249 | 19.73% |
| Obese (30+ kg/m^3^) | 803 | 9.15% | 194 | 9.77% | 326 | 7.09% | 97 | 8.87% |  | 1,420 | 8.62% |
| **Fertility Treatment prior to conception of index child:** | | | | | | | | | | | |
| No | 9074 | 96.85% | 1994 | 96.28% | 4763 | 98.45% | 1170 | 100% | <0.0001 | 17001 | 97.37% |
| Yes | 295 | 3.15% | 77 | 3.72% | 75 | 1.55% | * | - |  | 459 | 2.63% |
| * Insufficient numbers in group to create estimate  The numerical values in this table have been created from an estimation of the population value. The estimation arose because of the need to account for the survey design. As the numbers are estimates, percentages will not necessarily add up to 100%. | | | | | | | | | | | |
